# Assessment of the Antioxidant Activity of Catechin in Nutraceuticals: Comparison between a Newly Developed Electrochemical Method and Spectrophotometric Methods

**DOI:** 10.3390/ijms23158110

**Published:** 2022-07-23

**Authors:** Irina Georgiana Munteanu, Constantin Apetrei

**Affiliations:** Department of Chemistry, Physics and Environment, Faculty of Sciences and Environment, “Dunărea de Jos” University of Galaţi, 47 Domneasca Street, 800008 Galaţi, Romania; georgiana.munteanu@ugal.ro

**Keywords:** catechin, laccase, biosensor, antioxidant activity, DPPH, ABTS, galvinoxyl, carbon nanotubes, gold nanoparticles

## Abstract

The analysis of antioxidants in different foodstuffs has become an active area of research, which has led to many recently developed antioxidant assays. Many antioxidants exhibit inherent electroactivity, and, therefore, the use of electrochemical methods could be a viable approach for evaluating the overall antioxidant activity of a matrix of nutraceuticals without the need for adding reactive species. Green tea is believed to be a healthy beverage due to a number of therapeutic benefits. Catechin, one of its constituents, is an important antioxidant and possesses free radical scavenging abilities. The present paper describes the electrochemical properties of three screen-printed electrodes (SPEs), the first one based on carbon nanotubes (CNTs), the second one based on gold nanoparticles (GNPs) and the third one based on carbon nanotubes and gold nanoparticles (CNTs-GNPs). All three electrodes were modified with the laccase (Lac) enzyme, using glutaraldehyde as a cross-linking agent between the amino groups on the laccase and aldehyde groups of the reticulation agent. As this enzyme is a thermostable catalyst, the performance of the biosensors has been greatly improved. Electro-oxidative properties of catechin were investigated using cyclic voltammetry (CV) and differential pulse voltammetry (DPV), and these demonstrated that the association of CNTs with GNPs significantly improved the sensitivity and selectivity of the biosensor. The corresponding limit of detection (LOD) was estimated to be 5.6 × 10^−8^ M catechin at the CNT-Lac/SPE, 1.3 × 10^−7^ M at the GNP-Lac/SPE and 4.9 × 10^−8^ M at the CNT-GNP-Lac/SPE. The biosensors were subjected to nutraceutical formulations containing green tea in order to study their catechin content, using CNT-GNP-Lac/SPE, through DPV. Using a paired *t*-test, the catechin content estimated was in agreement with the manufacturer’s specification. In addition, the relationship between the CNT-GNP-Lac/SPE response at a specific potential and the antioxidant activity of nutraceuticals, as determined by conventional spectrophotometric methods (DPPH, galvinoxyl and ABTS), is discussed in the context of developing a fast biosensor for the relative antioxidant activity quantification.

## 1. Introduction

Phenolic compounds are naturally occurring secondary metabolites found in fruits and vegetables with potential antioxidant, cardiovascular, and, in some cases, tumor-preventing properties. These compounds also have significant benefits resulting from their ability to neutralize most oxidizing molecules such as hydroxyl anions, hydrogen peroxide, superoxides and singlet oxygen [1].

They can be classified into different functional categories according to the number of phenolic rings they contain and on the basis of structural elements that link these rings together. Thus, they can be divided into four main categories, namely phenolic acids, flavonoids, stilbenes and lignans.

Flavanols belong to the flavonoid subclass and consist of two aromatic rings (A and B) linked by three carbon atoms forming a heterocyclic ring with six oxygen-containing atoms (C). The exceptional antioxidant properties that these compounds possess are due to the oxidation of the hydroxyl groups in ring B (catechol structure). Catechins or flavanol-3-ols make up a group of compounds containing about 70% of the total polyphenols present in tea leaves. The important catechins present in green tea or green leaf are epigallocatechin (EGC), epigallocatechin gallate (EGCG), epicatechin gallate (ECG), epicatechin (EC), (+)-catechin (+C) and gallocatechin (GC).

Catechin has remarkable medicinal value due to the antibacterial, antitumor, anti-inflammatory and anti-diabetic properties [2]. At the same time, this compound is recognized for its antioxidant properties, having the ability to neutralize free radicals [3].

Given the health benefits, the quantitative determination of catechin in various products has attracted increasing interest. This analysis has been carried out by various methods, including gas chromatography [4], high-performance liquid chromatography [5], chemiluminescence [6], mass spectrometry [7], thin layer chromatography [8,9] and capillary electrophoresis [10]. In general, these methods are highly sensitive and efficient. However, such analytical methods are usually performed in centralized laboratories, require extensive analytical resources and labor and are often expensive and time-consuming. It is therefore of great interest to establish a rapid, simple, sensitive and inexpensive method for the detection of catechin. Generally, electrochemical analysis has been proposed as an efficient alternative for the determination of this compound, taking into account the advantages of this technique: simple operation, low cost, increased sensitivity and selectivity and rapidity of the method. However, the electrochemical analysis of catechin using traditional electrodes may be influenced by electroactive or non-electroactive interfering species [11]. Thus, to improve the analytical response, bio-nanomaterial-based sensors can be used, these devices being characterized by high selectivity, fast response, low cost and simple set-up, without the need for sample pre-treatment steps or laborious instruments for analysis [12].

The antioxidant activity of catechin is due to the scavenging of free radicals via hydroxyl groups. In general, antioxidant molecules can deactivate radicals by two major mechanisms: hydrogen atom transfer (HAT) and single electron transfer (SET) [13]. Depending on the mechanism involved, methods for determining the antioxidant activity can be classified as methods based on the HAT mechanism (the oxygen radical absorbance capacity (ORAC) test or total reactive antioxidant potential (TRAP) test) or methods based on the SET mechanism (ferric ion reduction test (FRAP); 2,2-diphenyl-1-picrylhydrazyl radical (DPPH) test; or 2,2-azinobis-3-ethyl-benzothiazolin-6-sulfonate (ABTS) cation radical test). The main difference between the two mechanisms is that the former evaluates the hydrogen atom transfer reaction, while the latter quantifies the reducing activity of an antioxidant [14].

There are, however, a number of drawbacks of these spectrophotometric methods that limit their use for rapid screening of antioxidant activity. These drawbacks are related to the need for a large amount of sample and reagents [15], the fact that they are laborious, time-consuming manual processes [16] and the necessity, in certain situations, of special analytical conditions [17] (e.g., analysis in the dark, incubation at 50 °C on a water bath, etc.).

Since radical scavenging is an electron transfer reaction, electrochemical methods can be successfully used to evaluate the antioxidant behavior of compounds, solving these limitations of colorimetric methods. For electrochemical oxidation, the major advantage is related to the previously established working conditions (e.g., a certain potential at which the oxidation reaction will take place [18]). It is also well known that there is an inverse proportional relationship between the oxidation potential and the electrochemical potential (higher antioxidant power corresponds to lower potentials [19]). Among electrochemical techniques, cyclic voltammetry (CV) [20,21,22] and differential pulse voltammetry (DPV) [23,24] are the most commonly used in various studies carried out to analyze redox systems.

Moreover, determining antioxidant activity through electrochemical sensors or biosensors has many advantages as compared to the conventional chemical methods, and it could be used for the initial screening of antioxidants. This technology does not require chemical reagents or solvents; neither does it require special treatment of samples. It offers extended and reproducible information about electrodynamic processes and a rapid achievement of determinations [25]. The development of electrochemical biosensors may be also suitable for catechin detection, thus taking advantage of the recognition properties of an enzyme immobilized on the surface of an electrode.

An enzyme active toward the ortho and para-diphenol groups, including mono-, di- and polyphenols, aminophenols or methoxyphenols, is laccase (Lac) [26]. Lac was used in biosensors based on metallic nanoparticles [27], carbon nanomaterials [28], polymers, and various membranes such as Nafion [29] and chitosan [30]. Of great importance is the synergistic combination of these nanomaterials and Lac, which increases the performance of electrochemical biosensors, in terms of sensitivity and selectivity [31]. It should also be pointed out that, from among the redox enzymes, Lac has a very good stability, which makes it ideal for antioxidant analysis.

Therefore, the aim of this study is to develop easy-to-use enzyme sensors with fast response and high accuracy applied to catechin detection in different nutraceutical formulations containing green tea extract. These new sensors were obtained by Lac enzyme modification of three electrodes as follows: a screen-printed electrode modified with carbon nanotubes (CNT/SPE), a screen-printed electrode modified with gold nanoparticles (GNP/SPE) and a screen-printed electrode modified with carbon nanotubes and gold nanoparticles (CNT-GNP/SPE). The electroanalytical method, developed in the present study, proved to be effective, due to the advantages it presented: precision, accuracy, simplicity, portability and low cost. This method can be used to control the quality of nutraceutical formulations, and may be extended to the analysis of catechin in other types of samples, such as food, beverages and cosmetic products.

Also, the most important objective of this study was the evaluation of antioxidant activity of these nutraceutical formulations containing green tea extract by means of the proposed electrochemical methods. Because of the differences between reaction mechanisms of different assays, a single assay will not reflect all the antioxidants present in a sample. Therefore, five methods were used for appreciation of antioxidant activities: two electrochemical methods (CV and DPV) and three SET-based chemical assays, namely DPPH, ABTS and galvinoxyl. Finally, a correlation was made between the data obtained by the electrochemical methods and those obtained by spectrophotometric ones.

## 2. Results and Discussion

### 2.1. Characterization of the Biosensors

The construction of a robust biosensor involves obtaining analytically important parameters such as lifetime, sensitivity, detection and quantification limits, each of which is directly related to the immobilization of the enzyme on the electrode surface.

Two methods, namely electrochemical impedance spectroscopy (EIS) and Fourier transform infrared (FTIR) spectroscopy, were used to observe the changes of the three laccase-based biosensors. In the case of CNT-GNP-Lac/SPE, the surface morphology was studied by scanning electron microscopy (SEM).

#### 2.1.1. EIS Study for CNT-Lac/SPE, GNP-Lac/SPE and CNT-GNP-Lac/SPE

To clarify the differences in the electrochemical performance of modified electrodes, EIS was used as a technique for electrochemical characterization of their surfaces as well as the charge transfer resistance for each immobilization, which indicates the interaction of the enzyme with the substrate [32]. The impedance (Z) represents the total resistance that the circuit offers to the alternating current flow at a given frequency [33].

**Figure 1:**
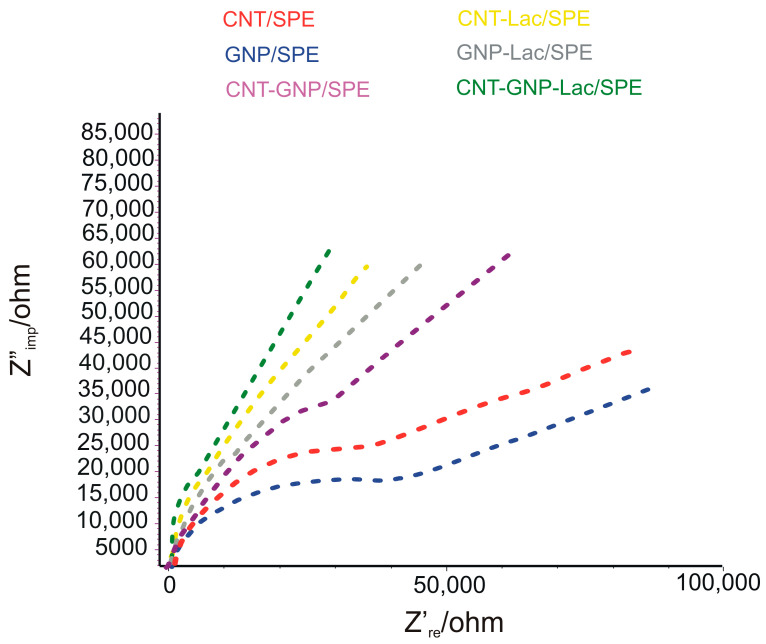
Nyquist plots for 1 mM [Fe(CN)_6_]^3−^ and 1 mM [Fe(CN)_6_]^4−^ obtained at CNT-Lac/SPE, GNP-Lac/SPE and CNT-GNP-Lac/SPE, in 10^−1^ M KCl, open circuit mode, 10 mV amplitude and frequency range of 100 MHz–100 kHz.

Figure 1 shows impedance spectra in the form of Nyquist plots in which the imaginary impedance (Z_im_) is plotted against the real impedance (Zre) as a function of frequency for 1 mM [Fe(CN)_6_]^3−^ and 1 mM [Fe(CN)_6_]^4−^ at all unmodified electrodes including CNT/SPE, GNP/SPE, CNT-GNP/SPE and Lac-modified electrodes including CNT-Lac/SPE, GNP-Lac/SPE and CNT-GNP-Lac/SPE in 10^−1^ M KCl.

The Randles circuit was selected to fit the experimental data obtained by EIS, which is presented in Figure 1, inset. This circuit comprises a solution resistance (*Rs*), a charge transfer resistance (*R*ct), a Warburg impedance (Z*W*) and a double layer capacitance (*C*dl) [34]. In impedance graphs, the semicircle section with respect to the electron transfer limited process and its diameter is related to the electron transfer Rct that controls electron transfers kinetics of redox probe at the electron interface [32].

Figure 1 shows the typical EIS curves of CNT/SPE, GNP/SPE and CNT-GNP-/SPE electrodes, as well as CNT-Lac/SPE, GNP-Lac/SPE and CNT-GNP-Lac/SPE electrodes in 10^−1^ M KCl solution containing 1 mM K_3_Fe(CN)_6_ and 1 mM K_4_Fe(CN)_6_. The semicircle portion at higher frequencies corresponds to the electron transfer limited process, and the linear portion at lower frequencies corresponds to the diffusion process. The respective semicircle diameters at the high frequency equal the Rct at the electrode surface [35].

It was found that the Rct of the CNT-Lac/SPE was about 4074 Ω, which was smaller than 32,500 Ω of CNT/SPE. Also, the Rct of the GNP-Lac/SPE was about 7350 Ω, smaller than 37,300 Ω of GNP/SPE. Finally, it can be appreciated that the adsorption of Lac on the surface of CNT-GNP-Lac/SPE is directly related to the decrease of the semi-circle and the increased electron transfer (880 Ohm) due to the coating of the electrode surface, confirming the presence of a new conductive layer, thus demonstrating the GNPs present in the CNT film enhance the electron transfer between the reactant and the electrode surface [36].

#### 2.1.2. FTIR Spectrophotometric Method

**Figure 2:**
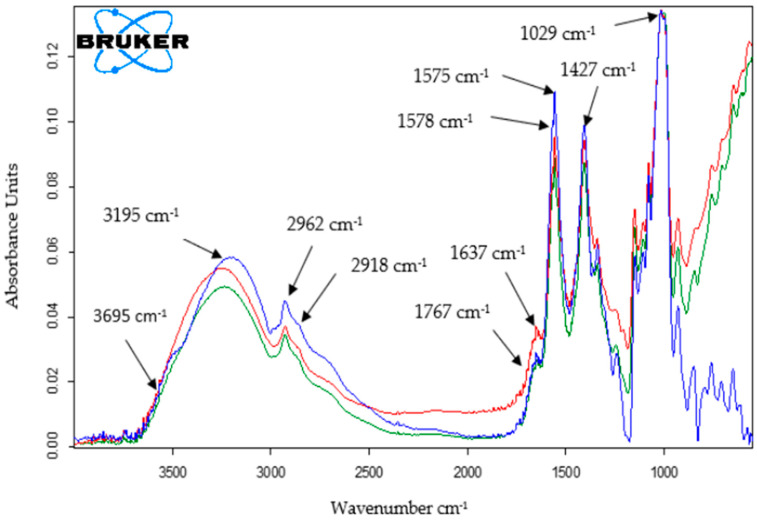
FTIR spectra for CNT-Lac/SPE (red line), GNP-Lac/SPE (green line) and CNT-GNP-Lac/SPE (blue line).

The second method used to characterize the three sensors was the infrared spectrometric method and the results are shown in Figure 2.

Several peaks representative of the presence of Lac are indicated by two bands at 1637 cm^−1^ and 1578 cm^−1^, which are respectively attributed to the secondary amide bond (C=N bond) of laccase with glutaraldehyde. In addition, C-H stretching vibration at 2962 cm^−1^ in all traces arises from the -CH_2_- group of glutaraldehyde [37].

Characteristic laccase peaks can be easily observed at the following wavenumber values: 3695 cm^−1^ corresponding to O-H group stretching vibrations (ν (O-H)) [38], 2918 cm^−1^ corresponding to ν (C-H) [39], 1767 cm^−1^ attributed to ν (C=O) [40], 1575 cm^−1^ characteristic for ν (C=C) [39], 1427 cm^−1^ characteristic for ν (C-N) [41] and 1029 cm^−1^ assigned to ν (C-O) [42].

In the FTIR spectrum of CNT-GNP-Lac/SPE, the typical absorption bands were very similar to those in the spectrum of GNP-Lac/SPE.

In all three cases, the absorption corresponding to the range 3000–3700 cm^−1^ is attributed to the elongation vibration of hydroxyl groups [43].

#### 2.1.3. Morphological Characterization through SEM

**Figure 3:**
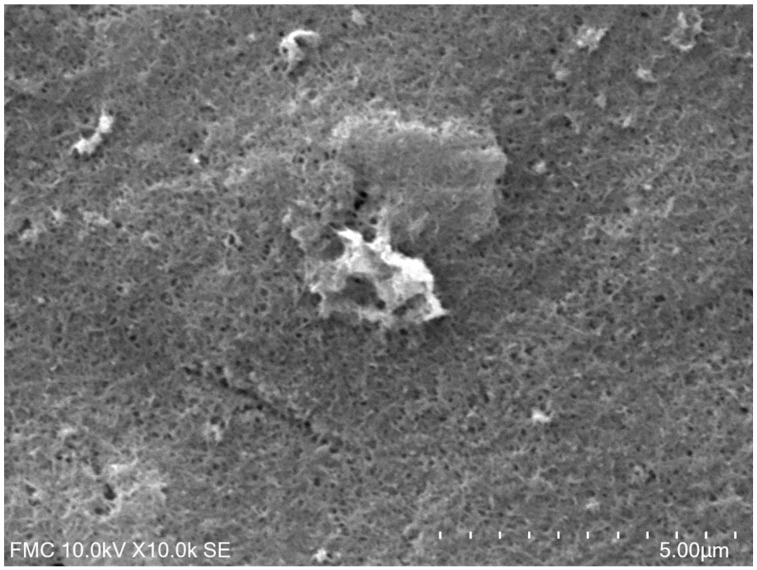
Scanning electron micrograph of the active surface of CNT-GNP-Lac/SPE.

Figure 3 shows the scanning electron microscope image highlighting the surface morphology of the composite nanofilm containing CNTs, GNPs and the enzyme Lac. As can be observed in Figure 3, CNTs were randomly oriented and solely distributed without wrapping with each other on the SPE surface and GNPs were uniformly dispersed without obvious aggregation. The Lac is also visible on the biosensor active surface.

### 2.2. The Influence of pH on the Performance of Biosensors

The catalytic activity of Lac extends between a strongly acidic and slightly basic environment, so pH optimization is a key factor for biosensitivity. pH changes also affect the protonation mechanism involved in the electrochemical redox reaction of phenolic compounds. According to specialized studies, it has been found that the optimal pH value for the detection of phenolic compounds is 5.0 [37]. The peaks obtained at this pH value are more obvious and well-defined. In addition, a lower pH value could contribute to the faster degradation of the enzyme [44].

**Figure 4:**
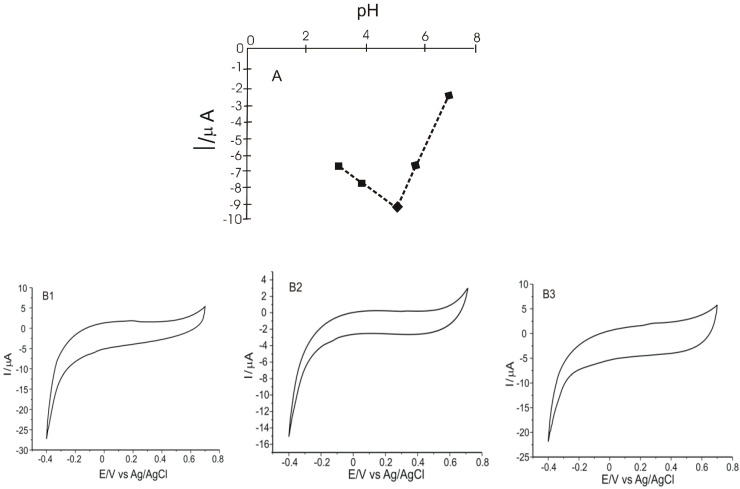
pH influence on the cathodic peak current intensity (**A**); Cyclic voltammograms of CNT-Lac/SPE (**B1**), GNP-Lac/SPE (**B2**) and CNT-GNP-Lac/SPE (**B3**) immersed in 10^−1^ M acetate buffer, pH = 5.2.

Thus, in order to establish an optimal pH value at which further determinations will be carried out in this study, the electrochemical behavior of the three biosensors in 10^−1^ M acetate buffer with different pH values (3.0, 4.0, 5.0, 6.0) was evaluated, at a scan rate of 0.1 V·s^−1^. When CNT-Lac/SPE, GNP-Lac/SPE and CNT-GNP-Lac/SPE were immersed in a 10^−1^ M acetate buffer at various pH values, the cyclic voltammograms showed, in all cases, two peaks: an anodic one of low intensity and a cathodic one that is more obvious. It can be clearly observed that for a pH higher than 6.5, the response decreases dramatically, and a maximum response is reached at about pH 5.2. At this pH value, the anodic peaks occur at 0.19 V, 0.38 V and 0.31 V and the cathodic peaks occur at −0.11 V, −0.10 and −0.15 V in the case of CNT-Lac/SPE, GNP-Lac/SPE and CNT-GNP-Lac/SPE, respectively. The peaks are related to the electrochemical reducing process of Lac on the surface of the modified electrodes. Figure 4 shows the influence of the pH on the Lac reduction process on the electrode surface (Figure 4A) and the cyclic voltammograms of CNT-Lac/SPE, GNP-Lac/SPE and CNT-GNP-Lac/SPE immersed in 10^−1^ M acetate buffer, pH = 5.2 (Figure 4B1–B3).

This electrochemical behavior shows that Lac activity is optimal at a rather acidic pH, which is confirmed in other studies [45,46]. In these experiments, we confirmed that at this pH value, the activity of Lac was not negatively affected, its immobilization being performed accordingly. At pH = 6.5, there is a decrease in current, which is probably due to loss or inactivation of the enzyme activity.

### 2.3. Electrochemical Behavior of Electrodes in Catechin Solution

The qualitative and then quantitative determination of catechin was carried out by CV and DPV, these methods being useful for the interpretation of processes occurring at the electrode surface. In the case of CV, the scan rate used was 0.1 V·s^−1^.

**Figure 5:**
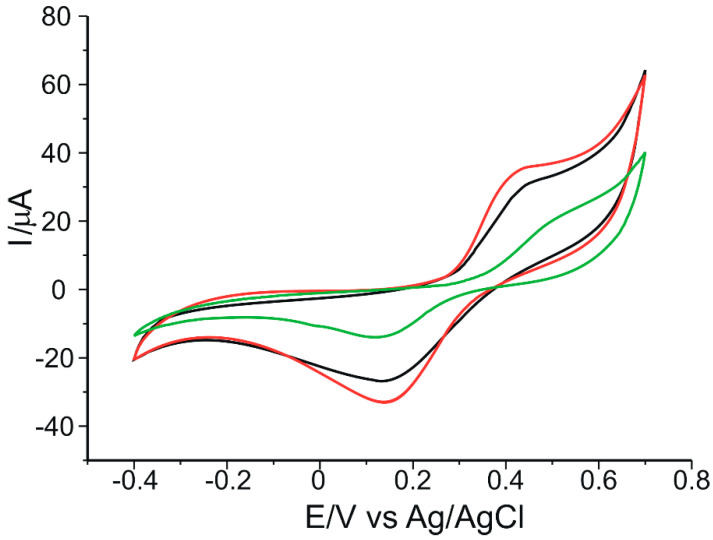
Cyclic voltammograms of 10^−3^ M catechin at a CNT-Lac/SPE (black trace), a GNP-Lac/SPE (green trace) and a CNT-GNP-Lac/SPE (red trace) in 10^−1^ M acetate buffer (pH 5.2). Scan rate: 0.1 V·s^−1^.

Figure 5 shows the cyclic voltammograms of 10^−3^ M catechin at the three electrodes in 10^−1^ M acetate buffer (pH 5.2). In order to obtain a stable sensor response, three cycles in the optimized potential range (−0.4 V to 0.7 V) were required.

**Figure 6:**
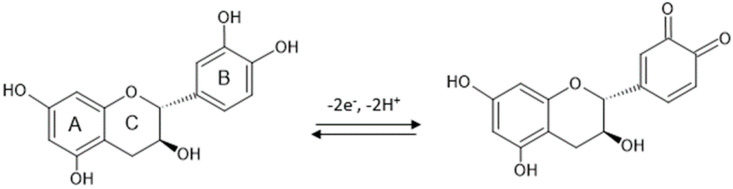
The mechanism of reversible oxidation of catechin.

In all three cases, an anodic and a cathodic peak of different intensities, but of similar potentials, corresponding to the oxidation or reduction of catechin at the aromatic B-ring level (Figure 6), respectively, are evident. This can be explained by the fact that there are two different groups in the structure of catechin–the catechol group in the B ring and the resorcinol group in the A ring–as well as the hydroxyl group in position 3 in aromatic ring C (Figure 6). The A and B rings of catechin are not conjugated, and ionization of the OH groups of one ring system should not significantly affect the ionization of the OH groups of the other aromatic ring [47]. Therefore, the ionizations of the OH groups of ring A are independent and distinct from those of ring B. Electron transfer occurs selectively to the aromatic cycle with the lower redox potential, which, in this case, is ring B [48]. Thus, the peaks highlighted in the voltammograms are the peaks corresponding to the reduction of the corresponding catechol group (the 3′,4′-dihydroxyl groups of ring B).

Previous studies containing theoretical calculations of the stability of the various catechin radicals have confirmed these trends: the 4′-phenoxyl radical was the most stable radical, and the other radicals were ordered in terms of their values characterizing electron affinity in the following sequence: 4′-OH, 3′-OH, 7-OH, 5-OH [49].

Figure 6 shows the oxidation mechanism of catechin, with the formation of the respective quinone.

In the voltammogram obtained at the CNT-GNP-Lac/SPE, the oxidation peak is 15% and 74% higher than that at the CNT-Lac/SPE and the GNP-Lac/SPE, respectively. Similarly, in the same voltammogram at the CNT-GNP-Lac/SPE, the reduction peak is 29% and 146% higher than that at the CNT-Lac/SPE and the GNP-Lac/SPE, respectively. This can be attributed to the synergistic effect of the association of CNTs with GNPs. On the one hand, CNTs exhibit good mechanical strength, excellent conductivity and remarkable electrocatalytic capacity [50], facilitating electron transfer for proteins or enzymes [51], and are unique due to the strong intermolecular bonds between the alternating hexagonal rings that lead to a crowded structure [52]. Moreover, recent publications have demonstrated that the modification of CNT electrodes facilitates electrochemical processes involving biomolecules and increases the measured signal [31,53,54]. On the other hand, GNP exhibit excellent electrical conductivity characteristics [55] (having unique chemical and physical properties, thus showing widespread use particularly for constructing electrochemical biosensors with a high electron transfer ability between the biomolecules and the electrode surface [56,57,58,59]), favorable biocompatibility [60,61], high specific surface area, which provides a stable immobilization of various biomolecules that thus retain their bioactivity [62,63], and, at the same time, a controllable particle size range, i.e., Jana et al. prepared the AuNPs with diameters of 5–40 nm by varying the ratio of seed to gold salt [64], whereas Rodriguez-Fernandez et al. prepared the AuNPs with diameters from 12 to 180 nm by incorporating small gold clusters on the surface of seed particles [65]. Bastus et al. reported a kinetically controlled seeded growth method for the synthesis of monodispersed citrate-stabilized AuNPs, with a uniform quasi-spherical shape of up to ∼200 nm, via the reduction of HAuCl_4_ by sodium citrate [66]. Recently, Riedel et al. synthesized spherical, silica-coated AuNPs, with an average diameter of 9 nm and a coating thickness of 2 nm, by improved pulsed laser ablation in liquid (PLAL), with this method offering great progress to the large-scale production of nanoparticles [67].

Therefore, a CNT-GNP-Lac/SPE provides a large specific surface area, resulting in a remarkable improvement of the reduction peak current. This large specific surface area can also accelerate electron transfer on the electrode surface to amplify the electrochemical signal and improve catechin performance on the modified electrode. Thus, CNT-GNP-Lac/SPE can provide an electron transfer microenvironment to facilitate the electrochemical reaction of catechin.

The electrochemical parameters obtained from the cyclic voltammograms of 10^−3^ M catechin at the three modified biosensors are shown in Table 1.

The half-wave potential (E_1/2_) is a qualitatively important characteristic for the electroactive species under analysis, expressed as the potential value for which the current strength is half of the maximum value [63]. As tabulated in Table 1, very similar E_1/2_ values are obtained for all three sensors. The Ipc/Ipa ratio is close to the ideal value of 1 in all three cases, the closest value being obtained in the case of CNT-GNP-Lac/SPE (1.01). Taking into account this value, but also the fact that for this modified sensor the difference between anodic and cathodic peak potentials (ΔE) is nearest to 29.5 mV, it can be stated that CNT-GNP-Lac/SPE has the highest degree of reversibility. Also, for this sensor the highest currents were obtained, followed by CNT-Lac/SPE and then GNP-Lac/SPE. From these results it can be concluded that the highest sensitivity for catechin detection was obtained for CNT-GNP-Lac/SPE.

From the study of the influence of the scan rate in the biosensors responses it was determined that the anodic and cathodic peak currents increase when the scan rates increases. In all cases, linear dependences between the cathodic peak currents and the scan rates were observed (Figure S1).

The biocatalytic mechanism of Lac on catechin as a substrate (S) is correlated with the active center of the enzyme containing four copper atoms (Type 1 (T1), Type 2 (T2) and Type 3 (T3)) [68]. In the T1 center, the copper atom is connected to two histidine (His) residues and two sulfurs from different sulfur-containing amino acids, such as cysteine (Cys) and leucine (Leu). The T1 center is characterized by a high redox potential and is therefore the main site where oxidation of many phenolic substrates (having a lower redox potential than the T1 center) occurs. The copper atom in the T2 center is coordinated with the two His residues. The T3 center is binuclear and contains two copper atoms, connected by anti-ferromagnetic force [69].

**Figure 7:**
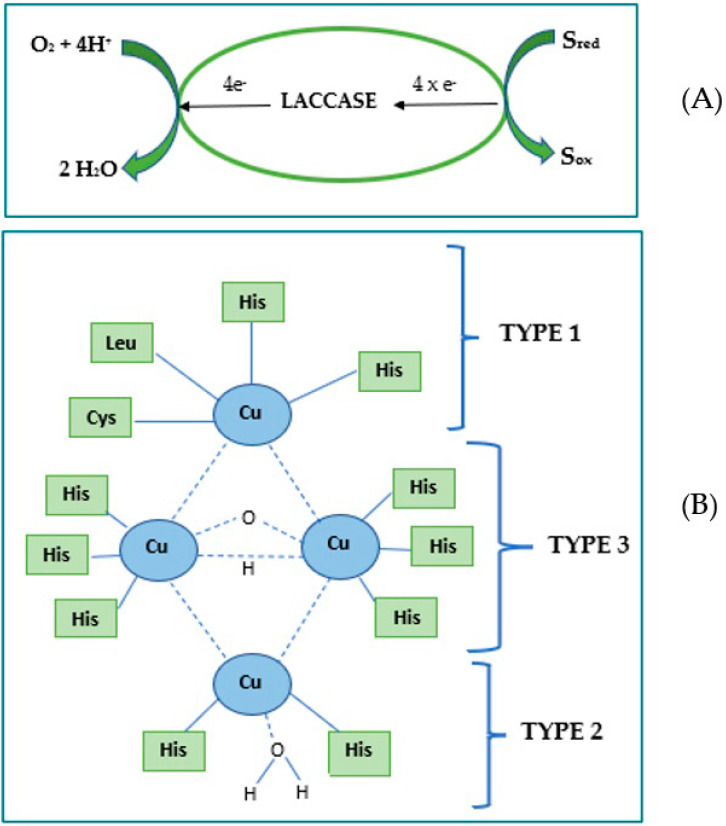
The oxidation mechanism of the Lac on a suitable substrate (**A**); Structure of the laccase active site Type 1, Type 2 and Type 3 (**B**).

In the first stage of Lac reaction mechanism, an electron is donated to the substrate by the copper T1 site, followed by an internal electron transfer from the reduced copper T1 sites to the copper T2 and T3 sites [70]. Copper T3 functions as a two-electron acceptor in the aerobic oxidation process, in which the presence of copper T2 is required [71]. The reduction of oxygen to water occurs at the T2 and T3 sites, passing through a peroxide intermediate (Figure 7) [72].

Therefore, Lac has the ability to catalyze the catechin oxidation process. Under the presence of air, o-hydroxylation of catechin will be catalyzed to o-diphenols, and the oxidation of o-diphenols will further be catalyzed to o-quinones [73]. The quinones formed are highly reactive and can undergo nucleophilic attack by other phenolic groups, amines, proteins and peptides [74]. Poly-catechin presents a much higher superoxide scavenging activity than the monomer catechin, making enzyme-catalyzed oxidative polymerization of phenolic compounds an important approach for producing new substances with higher antioxidant properties [75].

**Figure 8:**
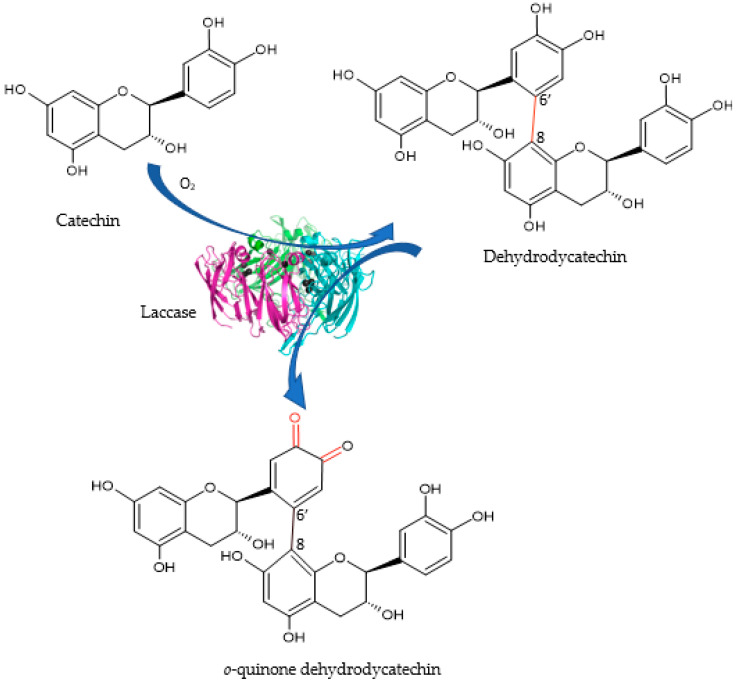
Schematic diagram of laccase-catalyzed catechin redox process.

The schematic diagram of laccase-catalyzed catechin is shown in Figure 8.

Since all three modified electrodes showed similar electrochemical behavior according to the parameters obtained, they were successfully used in subsequent determinations.

Thus, to study the behavior of the three modified sensors in 10^−3^ M catechin solution, we also used the DPV technique. To perform electrochemical measurements, the operational parameters were optimized to obtain good peak shape and high currents. The potential range used was −0.4 to 0.7 V, the pulse height was 0.10 V, the pulse width 0.5 s and the scan increment 0.01 V.

**Figure 9:**
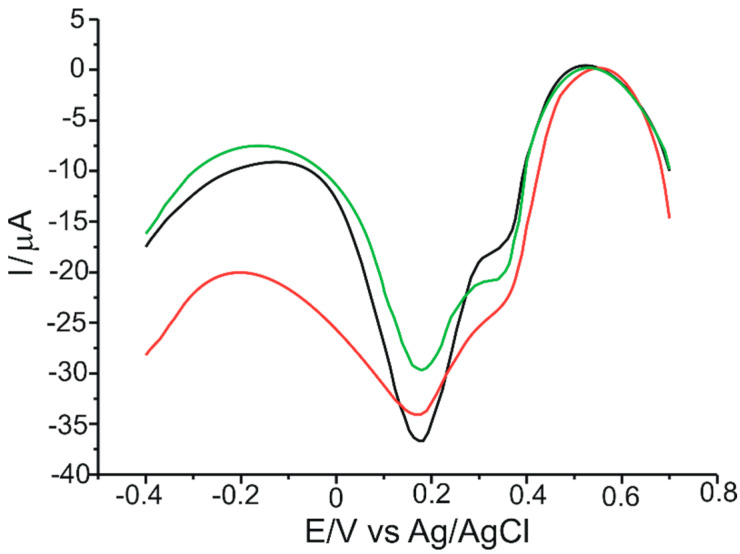
Differential pulse voltammograms for 10^−3^ M catechin at a CNT-Lac/SPE (red trace), a GNP-Lac/SPE (green trace), and a CNT-GNP-Lac/SPE (black trace) in 10^−1^ M acetate buffer (pH 5.2). The potential was scanned from −0.4 V to 0.7 V with a pulse height of 0.10 V, a pulse width of 0.5 s and a scan increment of 0.01 V.

Figure 9 shows the differential pulse voltammograms for the oxidation of 10^−3^ M catechin at a CNT-Lac/SPE, a GNP-Lac/SPE, and a CNT-GNP-Lac/SPE in 10^−1^ M acetate buffer (pH 5.2) as a supporting electrolyte. The potential range used was from −0.4 to 0.7 V.

DPV method achieves a higher resolution and enables improved peak separation to characterize subsequent steps in the electrooxidation. E_1/2_ of a peak in a cyclic voltammogram corresponds to the potential of a peak occurring in a differential pulse curve and is characteristic for each of the subsequent steps in the investigated electrode reaction. Voltammograms in Figure 9 show that catechin is oxidized irreversibly in two stages in the range of electrode potentials lower than the decomposition potential of the electrolyte. In the case of CV, peaks corresponding to the second stage of electro-oxidation of catechin does not exist. As determined by CV, E_1/2_ is 0.301 V, 0.328 V, 0.307 V in the case of CNT-Lac/SPE, GNP-Lac/SPE and CNT-GNP-Lac/SPE, respectively, corresponding to the peak potential of the second stage of DPV electrooxidation, at 0.387 V, 0.380 V and 0.392 V, respectively for the three modified biosensors.

### 2.4. Calibration Curve

In the next step involving quantitative determinations, the DPV voltammetric method was used, optimizing the parameters so as to obtain high currents and well-defined peaks.

**Figure 10:**
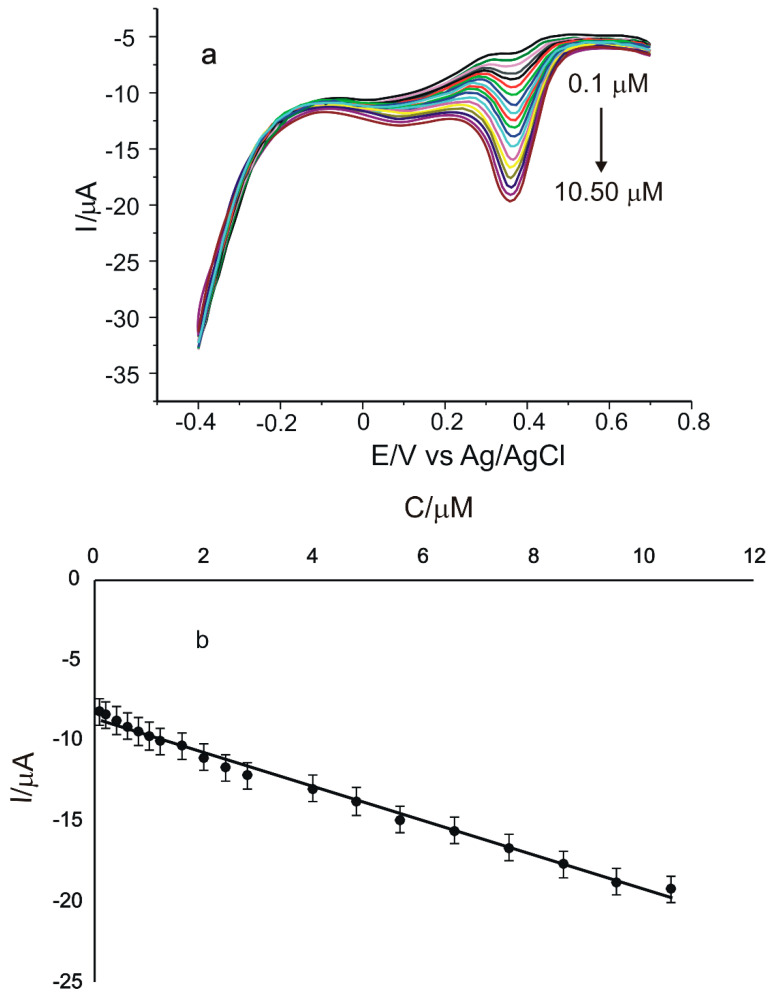
DPVs recorded for CNT-GNP-Lac/SPE in the concentration range of 0.1–10.50 µM catechin (**a**); linear fitting within the range of 0.1–10.50 µM for CNT-GNP-Lac/SPE (**b**). The potential range used was −0.4 to 0.7 V, the pulse height was 0.10 V, the pulse width 0.5 s and the scan increment 0.01 V.

The calibration curve for the concentration range of 0.1 µM–10.50 µM catechin obtained through DPV method for CNT-GNP-Lac/SPE is shown in Figure 10.

The linear dependence between I and c, and the quality of the linear model, was validated using the analysis of variance (ANOVA). The number of the experimental points was 19. At a 95% confidence level, the significance (F) is 1.24 × 10^−19^, highlighting the quality of the linear model. The linear equation of the linear model is I = −(1.06 ± 0.04)c–(8.63 ± 0.22).

Limit of detection (LOD) and limit of quantification (LOQ) are two important performance characteristics for method validation. These were calculated using the equations LOD = 3σ/m; LOQ = 10σ/m [63], where σ is the standard deviation (SD) of the electrochemical signal for the blank solution at the potential corresponding to the catechin peak and m is the slope of the linear calibration plot.

Table 2 shows the results obtained for LOD and LOQ by the DPV method, calculated for all three modified sensors used in this study.

Due to the synergistic effect of the association of GNPs with CNTs [76], interaction with catechin is favored, with improved selectivity and sensitivity of the CNT-GNP-Lac/SPE biosensor, which shows better performances than the other two biosensors.

Table 3 compares the data on the determination of catechin by the method proposed in this paper and other techniques reported in the literature.

Taking into account the values mentioned in this table, we can say that the low values of the detection and quantification limits are in agreement with the values obtained by other types of sensors or biosensors able to determine catechin in real-life samples.

The results obtained by the method proposed in this paper showed that the developed biosensors are excellent devices for the sensitive and selective determination of catechin. They are characterized by lower LOD value, wider linearity range compared to most previously studied sensors, short analysis time and high specificity on the analyte of interest conferred by the presence of the enzyme Lac on the sensor surface. In addition, the CNT-GNP-based electrode had remarkable performance due to the favoring of fast electron transfer between its surface and catechin [81].

### 2.5. Enzyme Kinetics: Calculation of Maximum Reaction Rate and Michaelis–Menten Constant

To evaluate the characteristics of an enzyme in solution, the Michaelis–Menten model is the most widely used, where reaction rates are measured as a function of enzyme-like substrate concentration [82].

The apparent Michaelis–Menten constant ($K_{M}^{app}$), an indication of both enzyme affinity and enzyme substrate kinetic constants, is determined from the electrochemical Lineweaver–Burk form of the Michaelis–Menten equation:

$$\frac{1}{I}=\frac{1}{I_{max}}+\frac{K_{M}^{app}}{I_{max}\times \left[S\right]}$$

where: *I* is the cathodic current, *I_max_* is the steady-state current and [*S*] is the concentration of the substrate.

A Lineweaver–Burk plot (1/*I* vs. 1/[*S*]) leads to a linear dependence and gives values about $K_{M}^{app}$ (from the slope of the equation) and 1/*I_max_* (from the ordinate intercept) [83]. The value of $K_{M}^{app}$ describes the affinity of an enzyme for its substrate, a lower value of $K_{M}^{app}$ indicating stronger substrate binding, i.e., higher biocatalytic activity [84].

The values obtained for the three biosensors are shown in Table 4.

It is found that the $K_{M}^{app}$ values are close for the three biosensors but significantly different at a 95% confidence level. This fact is related to the role of the immobilization matrix in the biocatalytic properties of the enzyme. The lowest value was obtained for CNT-GNP-Lac/SPE. This suggests that the affinity of the laccase to the substrate is stronger for this biosensor, giving it higher sensitivity [85].

### 2.6. Stability, Selectivity and Repeatability of the CNT-GNP-Lac/SPE Biosensor

To demonstrate the stability of the CNT-GNP-Lac/SPE biosensor, 50 consecutive cycles were performed in a continuous scan in 10^−3^ M catechin in 10^−1^ M acetate buffer (pH 5.2) at a scan rate of 0.1 V·s^−1^. After 50 cycles, a relative standard deviation of 2.1% was observed in the cathodic peak current, demonstrating the excellent operational stability of the biosensor. Therefore, the proposed CNT-GNP-Lac/SPE electrode could be used for catechin detection with high repeatability and excellent stability, characteristics resulting from the improved biocompatibility and catalytic activity of CNT and GNP together with the Lac enzyme.

**Figure 11:**
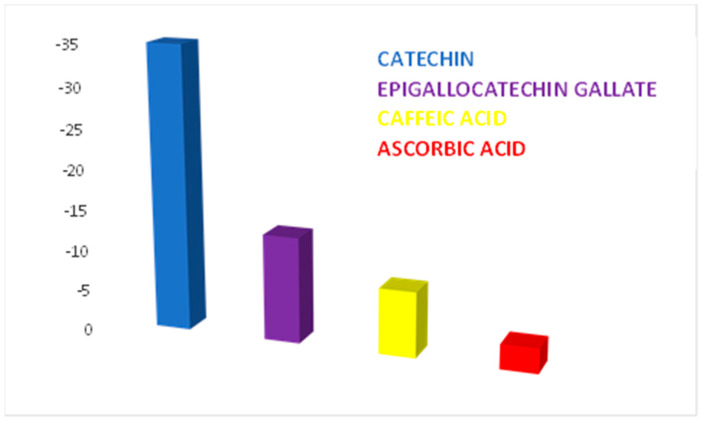
Selectivity of the CNT-GNP-Lac/SPE biosensor on the determination of catechin, epigallocatechin gallate, ascorbic acid and caffeic acid (electrolyte support: ABS 10^−1^ M, pH = 5.2). Scan rate: 0.1 V·s^−1^.

The biosensor selectivity was examined in the presence of other important constituents of green tea, namely epigallocatechin gallate (the most important component of the polyphenolic fraction of green tea), ascorbic acid and caffeic acid. The results are presented as a bar chart shown in Figure 11. The CNT-GNP-Lac/SPE electrode delivered a higher current intensity to its target analyte (catechin) than the other three analogue compounds.

The reproducibility of the proposed electrode was analyzed using CV. Three different sensors were modified using the same technique, subsequently recording the responses obtained in a solution of catechin 10^−3^ M-ABS 10^−1^ M. The electrodes demonstrated reasonable reproducibility, with the relative standard deviation value of the cathodic peak being 4.85%.

### 2.7. Quantitative Determination of Catechin in Nutraceuticals

In order to validate the biosensor in the catechin analysis from real-life samples, three different products from different manufacturers and containing catechin in different concentrations were selected and analyzed: Green Tea Adams Vision, Green Tea Extract Bio Synergie, Green Tea Extract Zenyth.

Green Tea Adams Vision is a product that helps to burn calories, lose weight and de-toxify the body. Green tea maintains the immune system and provides cardiovascular protection, reducing the risk of stroke.

Green Tea Extract Bio Synergie is an effective product with an antioxidant role through the substances it contains, including catechin considered more active than vitamin E.

Green Tea Extract Zenyth is a dietary supplement with standardized green tea leaf extract. The product is described as promoting antioxidant protection, supporting metabolism, strengthening immunity (immunomodulatory and anti-allergic), maintaining cardiovascular health, reducing inflammation, neuroprotective, supporting brain health and maintaining cholesterol and blood sugar levels within normal limits.

These products were quantitatively analyzed using the DPV electrochemical method. The aim of this analysis was to compare the results of the experiment and the values indicated by the manufacturers on the label of the nutraceutical products analyzed.

All nutraceutical products were analyzed using the CNT-GNP-Lac/SPE and the results obtained are discussed below.

**Figure 12:**
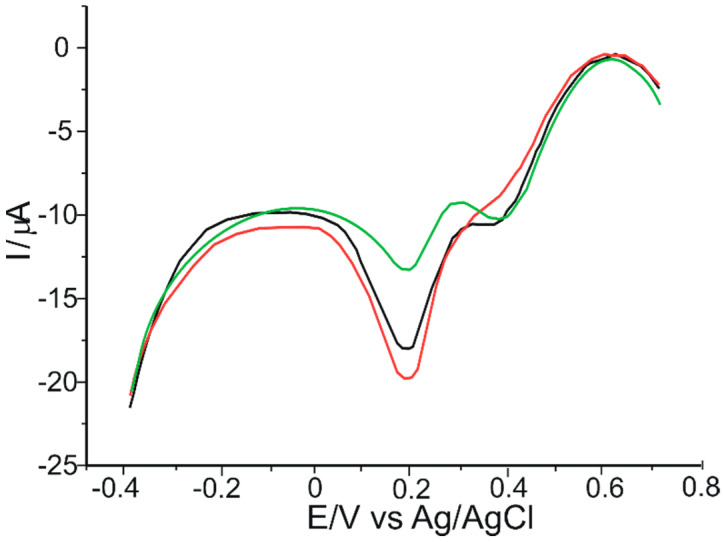
Differential pulse voltammograms of CNT-GNP-Lac/SPE immersed in a solution containing 9 mg product/50 mL ABS 10^−1^ M prepared from: Green Tea Adams Vision (black line), Green Tea Extract Bio Synergie (green line) and Green Tea Extract Zenyth (red line).

Figure 12 shows the voltammograms obtained for the three products studied, using solutions containing 9 mg product/50 mL ABS 10^−1^ M.

In all three voltammograms shown in Figure 12, the cathodic peak corresponded to the presence of catechin in the samples (on the basis of which this substance was quantitatively determined in the three products), and also the peaks corresponded to the presence of other compounds present in the nutraceutical formulations studied are observed.

Peaks corresponding to the presence of catechin occur at approximately the same potential values as those determined from the differential pulse voltammograms of the three biosensors immersed in a 10^−3^ M catechin solution (Figure 9).

Catechin amounts in nutraceutical formulations were determined by interpolation in the catechin calibration plot, obtained from the data obtained by DPV voltammetric method, of the peak current obtained by DPV in solutions of nutraceutical products. Dilutions and the amount of nutraceutical used in the analysis were taken into account for the calculations of the reported values (Table 5). All quantitative experiments were performed in triplicates. Results are reported as means of three replicates, being expressed in mg catechin per capsule.

As can be seen in Table 5, the results obtained using the DPV method as well as those provided by the producers are similar. The paired *t*-test assuming equal variances have shown that at 95% confidence level there are no significant differences between the means, which demonstrates the accuracy of the catechin quantification method presented in this study. Therefore, the biosensor could be successfully applied in laboratory practice in the quality control of pharmaceutical products containing catechin.

### 2.8. Evaluation of Antioxidant Activity by Spectrophotometric Methods

We have studied the ability of catechin to neutralize free radicals using DPPH, ABTS and galvinoxyl assays.

The use of the DPPH assay provides an easy and rapid way to assess antioxidants by spectrophotometry, and different products with antioxidant activity can be evaluated.

The galvinoxyl method is recommended for studies of hydrogen and electron yielding compounds, and is more sensitive to phenolic compounds than the DPPH method [86].

The ABTS method of assessing antioxidant activity is well known and widely used to determine the antioxidant activity of both pure substances and mixtures of compounds with antioxidant properties [87]. The additional advantage of the method is its applicability over a wide pH range [88]. Due to these advantages, the ABTS assay is used in numerous studies, allowing an easy, rapid and reliable determination of the antioxidant properties of the examined compounds [89].

These three methods are complementary and provide valuable information on the ability to react with free radicals. At the same time, all of these spectrophotometric methods were also applied to nutraceutical products in order to evaluate their antioxidant activity and to make a comparison with the antioxidant activity obtained from the pure substance alone.

The results obtained by the three methods, in the case of catechin, are reported as the average of 3 replicates for the percentage inhibition values for all standard solutions tested and are shown in Table 6.

In the case of the three nutraceuticals studied, the results for the percentage inhibition values are shown in Table 7.

It is noted that the results of the DPPH and galvinoxyl assays differ from those of the ABTS assay. This is probably related to the different type of reagents used and relative poor selectivity of ABTS in the reaction with hydrogen-atom donors (i.e., catechin) comparing with DPPH or galvinoxyl [90].

Nevertheless, in the case of the three pharmaceutical formulations, there is a remarkable difference in the values obtained for Green Tea Extract Zenyth by all three analytical methods. These results are in agreement with the values obtained in the quantitative determination of catechin in nutraceuticals, where the highest amount of catechin contained per capsule was also obtained for this product.

### 2.9. Determination of Antioxidant Activity by Electrochemical Methods

Apart from spectrophotometric assays, electrochemical methods, such as CV and DPV, offer alternative strategies for determining the antioxidant activity of different samples.

By means of voltammetric methods, the main electrochemical parameters (peak potential-Ep and peak intensity-Ip) can be obtained, thus giving insights into the presumed antioxidant activity of each sample analyzed.

#### 2.9.1. CV–Anodic Area

**Figure 13:**
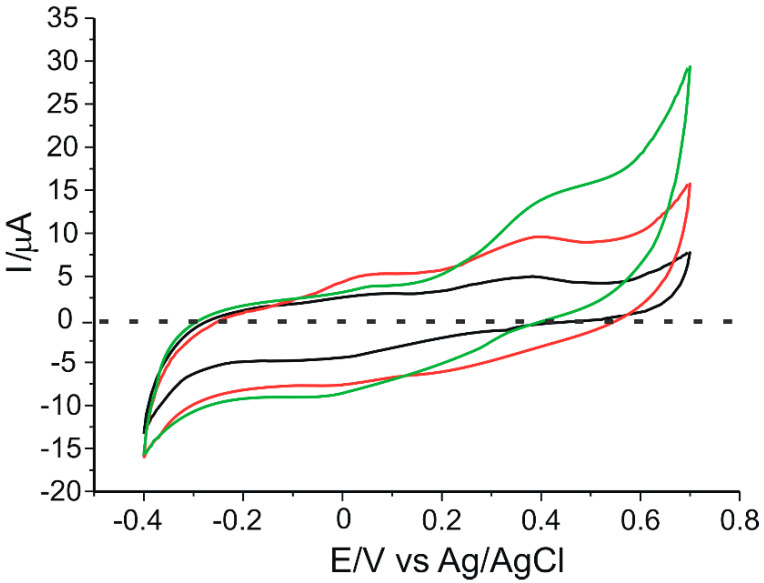
Cyclic voltammograms of 9 mg product/50 mL 10^−1^ M acetate buffer where the product is Green Tea Adams Vision (red trace), Green Tea Extract Bio Synergie (black trace) and Green Tea Extract Zenyth (green trace) at a CNT-GNPLac/SPE. Scan rate: 0.1 V·s^−1^.

A valid expression of the antioxidant activity of a given sample is evidenced by its redox profile [91]. According to the literature, the area under the anodic peak curve (Sa) can express the total reducing power of complex mixtures of antioxidant compounds, such as green tea extracts. Each anodic peak reflects a component or combination of components that donate electrons at approximately the same potential [92]. Therefore, the Sa of cyclic voltammograms of all three nutraceuticals recorded at a CNT-GNP-Lac/SPE can be correlated with the antioxidant activity of the respective products. Figure 13 illustrates the cyclic voltammograms of the three products studied, in the potential range −0.4 and 0.7 V, recorded at a scan rate of 0.1 V·s^−1^, using the CNT-GNP-Lac/SPE biosensor. Sa corresponds to the charge used in the experiment from the potential of 0.08 V to 0.4 V (Q400) and is used as a measure of the antioxidant content of the products.

In Figure 13, in all three voltammograms, the first peak which appears at 0.05 V may correspond to the presence of other flavonols in the studied products, while the second peak is associated with the presence of catechin, and it appears at approximately the same potential values (0.39 V) as those determined from the cyclic voltammograms of the biosensor immersed in a 10^−3^ M catechin solution (Figure 5).

Some features suggesting high antioxidant activity include the presence of electroactive species, which undergo electrochemical oxidation at anodic peak potential (Epa) bellow 0.5 V [14,19]. On the other hand, the higher the anodic peak intensity (Ipa), the higher the concentration of related species and/or kinetics in which the electron transfer would take place in a reduction reaction. Also, since the regenerating ability may improve the antioxidant function, the reversibility of a redox process is a remarkable behavior [93]. For instance, the presence of polyphenols presenting a catechol moiety, i.e., catechin in green tea extracts, combines the reversibility of catechol/quinone system with higher reducing activity associated to inherently low peak potentials, thus providing high antioxidant power [91].

Table 8 shows the values of the electrochemical parameters obtained from the cyclic voltammograms shown in Figure 13 and the Q400 values (expressed in µC) for the three pharmaceuticals studied. It is noted that the highest Q400 value was obtained for the Green Tea Extract Zenyth product, which can be attributed to the high amount of antioxidant compound, namely catechin, contained per capsule, compared to the other two products, as demonstrated earlier in this study by spectrophotometric methods.

Taking into account that antioxidants exert their reducing activity through electron transfer mechanisms (they undergo oxidation whereas protected species are reduced or kept unchanged from oxidizing agents), parameters are presented only for anodic curve (in the positive direction), where the phenolic compounds were acting as reducing agents.

From Table 8 it is evident that the product Green Tea Extract Zenyth has the highest Q400 value, which is in accordance with high catechin content (Table 5) and with the highest percentage of inhibition (Table 7), as previously demonstrated in this study.

Moreover, Q400 value correlates with the percentage inhibition values obtained by all three spectrophotometric methods, therefore electrochemical methods could be used for estimating the antioxidant activity of various nutraceuticals, dietary supplements, pharmaceuticals, etc.

#### 2.9.2. DPV-Electrochemical Index (EI)

The electrochemical index (*EI*) has been defined as a screening method meant to determine the total concentration of antioxidant compounds in different samples and can be obtained using electrochemical techniques, e.g., CV and DPV, taking into account the type of sample to be analyzed, *E_p_* and *I_p_*, using the following equation which defines the antioxidant capacity corresponding to the electrochemical properties of the respective samples [94]

$$EI=\left(I_{p1}/E_{p1})+(I_{p2}/E_{p2}\right)+\cdots +\left(I_{pn}/E_{pn}\right)$$

The thermodynamic parameter *E_p_* expresses the reducing power, while the kinetic and quantitative parameter *I_p_* expresses the electron transfer rate and/or the amount of antioxidant content in the sample [95].

To determine the *EI* of the three nutraceuticals studied, the values of the intensities and potentials obtained by the electrochemical DPV method (Figure 12) were taken into account. At the same time, the *EI* was also determined for pure catechin (based on differential pulse voltammograms presented in Figure 9) in order to compare the results. These are presented in Table 9.

For evaluation and analysis of the EI, the same factors previously observed for current and potential are taken into account. In other words, the lower the peak potential, the greater the electron donor capacity, and the higher the peak current, the higher the electron transfer and the number of electroactive species present in the samples. Thus, the higher the current values and the lower the potential values, the higher the EI values [96]. Therefore, the highest EI values were obtained for product Green Tea Extract Zenyth, approximately equal to that of the pure substance of concentration 10^−3^ M, which was expected since the peak current value obtained was higher (−20.80 V) and the peak potential value was lower (0.19 V) when compared to the other two products for which the following values were obtained: *I_p_*= −18.25 V; *E_p_* = 0.20 V in the case of Green Tea Adams Vision and *I_p_*= −12.60 V; *E_p_* = 0.21 V in the case of Green Tea Extract Bio Synergie.

Therefore, by comparing the results obtained by the three spectrophotometric methods and taking into account those obtained by electrochemical methods (CV and DPV), we can obtain information about the redox properties and radical scavenging activity induced by electron and hydrogen transfer [97] not only in the case of catechin alone, but also in the case of its determination in various nutraceutical products.

## 3. Materials and Methods

### 3.1. Reagents and Solutions

Three nanomaterial-modified electrodes, namely CNT/SPE, GNP/SPE and CNT-GNP/SPE, purchased from Metrohm DropSens (Oviedo, Spain), were used to obtain the biosensors, and all three were subsequently modified in the laboratory. This modification consisted in immobilization of the Lac enzyme followed by cross-linking, thus obtaining the three biosensors: CNT-Lac/SPE, GNP-Lac/SPE and CNT-GNP-Lac/SPE.

Lac from *Trametes versicolor* (0.78 U/mg) was purchased from Sigma-Aldrich and used without further purification. To immobilize the enzyme, a solution obtained from Lac of concentration 10 mg/mL in acetate buffer 10^−1^ M (pH = 5.2) was used.

Sodium acetate (NaCH_3_COO) of 99% purity and glacial acetic acid (CH_3_COOH) purchased from Sigma-Aldrich (St. Louis, MO, USA) were used to prepare the 10^−1^ M acetate buffer in Milli-Q (Millipore, Bedford, MA, USA) water. The pH was adjusted to 5.2 by the addition of hydrochloric acid, and the pH was measured using a pH meter from WTW Instruments, Weilheim, Germany.

Catechin of analytical purity was purchased from Sigma-Aldrich. A 10^−3^ M catechin stock solution was prepared using the 10^−1^ M acetate buffer (pH 5.2), which was used as the supporting electrolyte.

DPPH 0.1 mM stock solution was prepared with DPPH reagent (purchased from Sigma-Aldrich), dissolved in 96% (*v*/*v*) ethanol. The resulting solution was kept in the dark at room temperature.

The 0.1 mM galvinoxyl stock solution was prepared by initially dissolving the salt in 96% (*v*/*v*) ethanol, which was then kept in the dark at room temperature for 20 min, followed by the addition of 10^−3^ M catechin stock solution. The absorbance for each sample was measured at 860 nm [98]. The 96% (*v*/*v*) ethanol was used as reference.

To obtain the concentrated ABTS^·+^ cation radical solution, ABTS diammonium salt and potassium persulfate were dissolved in ultrapure water to a final concentration of 7 mM and 2.45 mM respectively. The solutions were mixed and stored in the dark for 20 h. ABTS^+^ cation radical solutions were diluted using 96% (*v*/*v*) ethanol.

### 3.2. Electrodes and Devices Used

Electrochemical measurements were performed using a conventional system containing three electrodes, namely an Ag/AgCl reference electrode (Princeton, Applied Research), an auxiliary electrode consisting of a platinum wire and a working electrode (CNT-Lac/SPE, GNP-Lac/SPE and CNT-GNP-Lac/SPE).

An EG&G potentiostat/galvanostat (Princeton Applied Research, Oak Ridge, TN, USA), model 263A, controlled by ECHEM software was used to characterize and optimize electrode signals and also for electroanalysis of nutraceutical formulations. The Partner AS 220/C/2 analytical balance was used for weighing substances and the Elmasonic ultrasonic bath (Carl Roth GmbH, Karlsruhe, Germany) for dissolving substances. The Inolab pH 7310 m was acquired from WTW Instruments, Weilheim, Germany.

FTIR spectra were acquired with a Bruker ALPHA FT-IR spectrometer (BrukerOptik GmbH, Ettlingen, Germany) using OPUS software (BrukerOptik GmbH, Ettlingen, Germany) in the 4000 to 500 cm^−1^ wavelength range, using the attenuated total reflectance (ATR) method as the sample exposure mode. The ZnSe crystal was carefully cleaned with ultrapure water and isopropanol between measurements. The background was the spectrum obtained in air.

For UV-Vis spectrophotometric method, absorbance was measured using a Rayleigh UV2601 UV/Vis double beam spectrophotometer (Beijing Beifen-Ruili Analytical Instrument, Beijing, China).

A scanning electron microscope (FlexSEM 1000 II, Hitachi, Japan) was used to analyze the surface morphology of the CNT-GNP-Lac/SPE biosensor.

### 3.3. Preparation of Biosensors

**Figure 14:**
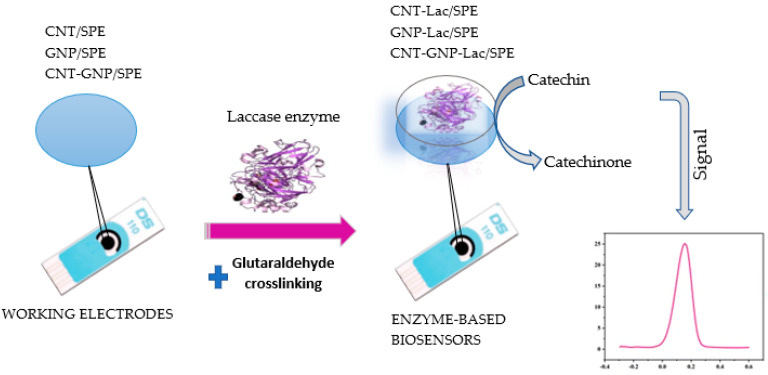
Preparation process of the laccase-based biosensor on the support of SPEs based on CNT, GNP and CNT-GNP.

Three sensors, namely CNT/SPE, GNP/SPE and CNT-GNP/SPE, were used as the substrate for biosensor preparation. As illustrated in Figure 14, using a *drop-and-dry* technique, 10 µL Lac enzyme solution was added to each sensor, sequentially in two steps (5 µL in each step) with a 3 h drying break between the two steps. Enzyme cross-linking was performed by suspending each sensor over 5 mL 2% (*v*/*v*) glutaraldehyde for 1 min. The glutaraldehyde vapor ensured immobilization of the Lac on the electrode surface by cross-linking. Biosensors were stored at 4 °C until use, for a maximum of 72 h [63].

### 3.4. Methods of Analysis

In the present work, two electroanalytical methods, including CV and DPV were used to study the oxidation-reduction processes taking place at the electrode surface and also to validate the results obtained.

#### 3.4.1. CV

CV was used to characterize working electrodes as well as the stage of catechin detection in the solution prepared with pure substance and in the solutions prepared with the real samples. The method is very suitable for these tests and provides valuable information on the electrochemical behavior of the substance under analysis [99]. The potential range was optimized, being between −0.4 and +0.7 V, and the scan rate varied from 0.1 to 1.0 V·s^−1^. CV was conducted using 10^−3^ M catechin solution.

#### 3.4.2. DPV

DPV is another widely used electrochemical technique suitable for characterizing the redox behavior of antioxidants [100].

DPV measurements were performed by sweeping working potential between −0.4 to 0.7 V with a scan rate of 0.05 V·s^−1^; the pulse height was 0.10 V, the pulse width 0.5 s and the scan increment 0.01 V.

It is well-known from the literature that CV is limited due to lower sensitivity if compared to pulse techniques, such as DPV [101]. The main advantage of DPV technique is the ability to detect the faradaic current (net current used in calibration/analytical curves) in the absence or minimal presence of the capacitive current [96]. Therefore, we selected both CV and DPV techniques to be used in subsequent experiments to evaluate the behavior of catechin on the modified surface of the electrodes. Moreover, the two methods have also been employed in determining the antioxidant activity of selected nutraceutical formulations containing green tea extract.

The results obtained with the two voltammetric techniques, CV and DPV, were compared and complementary information was obtained.

#### 3.4.3. EIS

For the electrochemical study of various electrodes (bare and modified) and evaluation of the electrochemical treatment of the stepwise modification of the electrode, EIS is a suitable method [102]. EIS was performed in a 10^−1^ M KCl solution containing 1 mM K_3_[Fe(CN)_6_]/K_4_[Fe(CN)_6_], with a (1:1) mixture at an open-circuit potential of 0.23 V. The alternating voltage was set at 10 mV, and the frequency range covered from 100 MHz to 100 kHz.

### 3.5. Real-Life Samples and Preparation of Testing Solutions

The nutraceutical capsule forms of all three green tea products were purchased from a health food store. The products have a diverse composition of active ingredients and excipients, and the package leaflet of each one states the presence of catechin, the substance of interest for this study.

The contents of one capsule of each drug product were dispersed in dispersed 50 mL of pH 5.2 acetate buffer for electrochemical analysis. An ultrasonic bath was used for homogenization and insoluble particles were separated by filtration.

### 3.6. Antioxidant Activity

Three methods, namely DPPH, galvinoxyl and ABTS, were used to determine the antioxidant activity of both pure catechin and nutraceuticals.

#### 3.6.1. DPPH Method

The spectrophotometric method based on the reaction of antioxidant products with the stable free radical DPPH (1,1-diphenyl-2-picrylhydrazyl) is widely used to measure the free radical neutralizing ability of a wide variety of antioxidants. 

**Figure 15:**
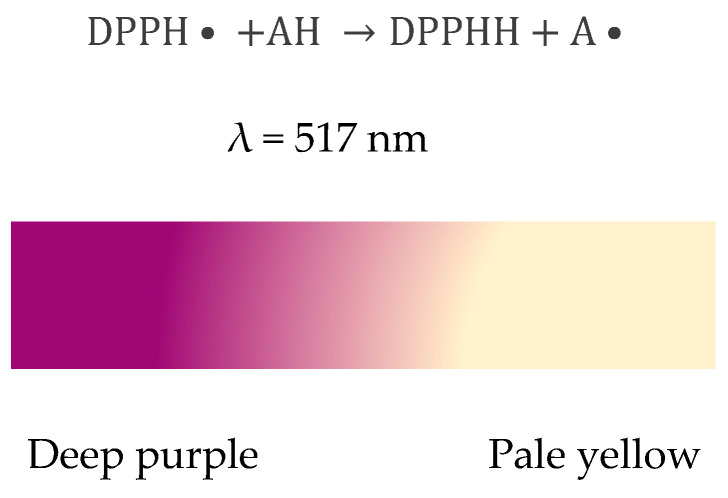
DPPH scavenging mechanism by an antioxidant.

The evaluation of antioxidant activity using this technique was performed according to the method previously described by Şenocak et al. [79]. The method involves recording the decrease in absorbance at 517 nm for DPPH· ethanolic solution following the addition of an antioxidant upon reaching steady state [72]. The reaction is accompanied by changing the DPPH color measured at 517 nm, and discoloration acts as an indicator of antioxidant activity (Figure 15).

In the first step of the determination, the 0.1 mM DPPH stock solution was prepared from the DPPH reagent and 96% ethanol and kept in the dark at room temperature. The test solution (catechin) had a concentration of 10^−3^ M. Volumes of 3 mL DPPH solution were measured in the spectrophotometer cuvettes, over which variable volumes of the catechin solution were added (the same volumes used for the determination of the calibration curve, between 5 µL and 520 µL). These were kept at room temperature for 20 min, after which the absorbances were measured at 517 nm against ethanol [73].

For DPPH analysis of nutraceuticals, sample solutions were obtained using the same amounts of products as used for electrochemical measurements. Volumes of 3 mL DPPH solution were measured in the spectrophotometer cuvettes over which 0.2 mL of product solution was added, and the absorbance for each sample was then measured at 517 nm after 20 min towards ethanol.

#### 3.6.2. Galvinoxyl Method

The galvinoxyl method of determining antiradical activity is based on the use of the stable O-centered radical galvinoxyl (GV^•^), which is known to associate with the physiological action of oxygen radicals rather than the stable N-centered radical of DPPH [103]. In this work, absorbance was measured at 860 nm. 

The same sensing mechanism of radical scavenging described in Figure 13 can be applied to the radical. However, the color change induced by exposure to antioxidants is not easily detected by the naked eye. The GV^•^ radical-scavenging reaction is given below:

$${\mathrm{GV}}^{•}+\mathrm{AH}\;\to \mathrm{GVH}+A^{•}$$

Galvinoxyl radical scavenging activity was evaluated according to the method described by Shi et al. [74]. A 0.1 mM galvinoxyl stock solution was prepared from galvinoxyl reagent and 96% ethanol and kept at room temperature in the dark. The analyte solution (catechin) had a concentration of 10^−3^ M. Volumes of 3 mL galvinoxyl solution were measured in the spectrophotometer cuvettes, over which variable volumes of the catechin solution were added, between 5 µL and 520 µL. These were kept at room temperature for 20 min, after which absorbances were measured at 860 nm [75] towards ethanol. 

For the application of the galvinoxyl method to the analysis of nutraceuticals, sample solutions were obtained using the same quantities of products as those used for electro-chemical measurements. Volumes of 3 mL galvinoxyl solution were measured in the spectrophotometer cuvettes over which 0.2 mL of product solution was added and the absorbance for each sample was then measured at 860 nm after 20 min towards ethanol.

#### 3.6.3. ABTS Method

The cation radical ABTS^•+^ is generated by oxidation of ABTS with potassium persulfate (K_2_S_2_O_8_) and is reduced by the addition of hydrogen atoms [104]. The principle of the method is based on lowering the absorbance of ABTS^•+^ [87]. This chromogenic compound shows a maximum absorption in the range 600–750 nm and can be easily determined spectrophotometrically [105].

**Figure 16:**
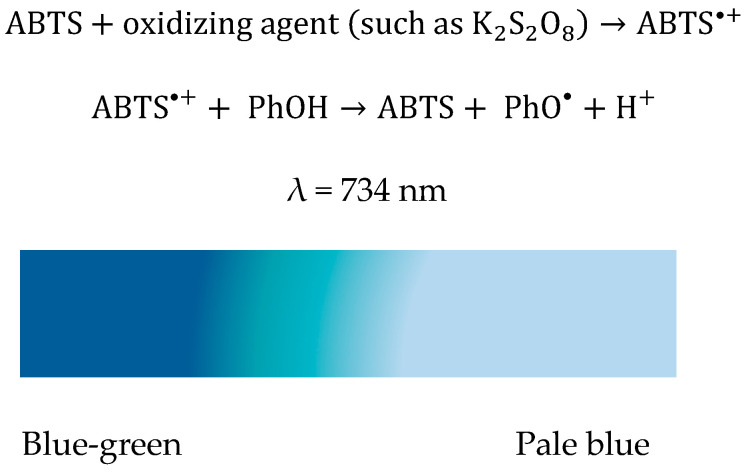
Reaction scheme involved in ABTS radical cation scavenging activity assay.

Antioxidant activity is measured as the ability of the test compound to decrease ABTS^•+^ color by intercepting initial oxidation and preventing ABTS^•+^ production or reacting directly with the preformed radical cation (Figure 16). Even when a fixed-time ABTS assay is preferred, the results may greatly vary for the same compound depending on the oxidizing agent used to generate the stable colored radical [106].

The ABTS^+•^ cation radical was prepared according to the method presented by Kurin et al. [76], with some adjustments. Equal volumes of 5 mM ABTS solution and 2.45 mM K_2_S_2_O_8_ solution were mixed, and the resulting mixture was kept at room temperature and in the dark for 24 h. After this time, the ABTS^+•^ cation radical solution was diluted with ethanol until the absorbance value at 734 nm was close to 1 [77]. Subsequently, volumes of 3 mL of the diluted ABTS solution were measured in the spectrophotometer cuvettes, to which varying volumes of the catechin solution, between 5 µL and 520 µL, were added. These were kept at room temperature for 6 min [78], and then absorbances were measured at 734 nm against ethanol.

In the analysis of nutraceuticals, 0.2 mL of each nutraceutical solution was mixed with 3 mL of diluted ABTS solution. The rection was allowed to react for 6 min, before the absorbance was measured at 734 nm.

For all three methods, the percentage reduction capacity of DPPH, galvinoxyl and ABTS radicals was calculated according to the following equation [107]:

$$\%\;\mathrm{Inhibition}=\left(\frac{\mathrm{AD}-\mathrm{AE}}{\mathrm{AD}}\right)\times 100$$

where AD is the absorbance of the control solutions and AE is the absorbance of the test solutions.

## 4. Conclusions

Catechin is an essential antioxidant and confers therapeutic properties to green tea, thus having a relevant impact on human health. In the present work, three Lac-based biosensors, namely CNT-Lac/SPE, GNP-Lac/SPE and CNT-GNP-Lac/SPE, were developed and characterized, with the CNT-GNP-Lac/SPE showing the best analytical performance with an LOD of 4.89 × 10^−8^ M and a sensitivity of 8.63 mA M^−1^. This can be attributed to the association of CNT with GNP, which increased the sensitivity of the biosensor significantly due to higher electroactivity as well as easier electron transport to the electrode surface. Three methods, namely EIS, FTIR and SEM, were used to characterize the surface of the modified electrodes. A linear range between 0.1–10.50 µM, an LOD of 4.89 × 10^−8^ M and an LOQ of 1.63 × 10^−7^ M were estimated using DPV method. The catechin content of 31.9, 16.8 and 203.4 mg/capsule, respectively, was determined in nutraceutical formulations using CNT-GNP-Lac/SPE in conjunction with DPV. These values are in agreement with those specified by the manufacturers.

This paper also brings together two types of methods, chemical (DPPH, galvinoxyl and ABTS) and electrochemical (CV and DPV), to characterize the antioxidant activity of catechin and of the three nutraceuticals studied. Thus, by means of the DPV voltammetric method it was possible to determine the electrochemical index of the pure compound and of the nutraceutical products, and by means of CV, Sa (correlated with the antioxidant activity) was evaluated. Both methods showed that the highest antioxidant activity was obtained in the case of Green Tea Extract Zenyth, being comparable to that of the pure compound, at a concentration level of 10^−3^ M.

The results obtained in this study are encouraging for the evaluation of the antioxidant activity of nutraceutical formulations containing catechin using voltammetric techniques. Compared to conventional methods, electrochemical methods are simple, fast, economical, reliable and do not require chemical reagents, and can be successfully used in screening assays for the evaluation of antioxidant compounds with applications in food and pharmaceutical fields.

## Figures and Tables

**Table 1:** The values of the parameters obtained from the cyclic voltammograms of 10^−3^ M catechin at electrodes in 10^−1^ M acetate buffer (pH 5.2) supporting electrolyte.

Biosensor	Epa ^1^ (V)	Ipa ^2^ (µA)	Epc ^3^ (V)	Ipc ^4^ (µA)	Ipc/Ipa	ΔE ^5^ (V)	E_1/2_ ^6^
CNT-Lac/SPE	0.467	29.76	0.136	−26.90	0.90	0.331	0.301
GNP-Lac/SPE	0.527	19.70	0.130	−14.08	0.71	0.397	0.328
CNT-GNP-Lac/SPE	0.464	34.20	0.150	−34.58	1.01	0.314	0.307

^1^ Potential of the anodic peak; ^2^ current of the anodic peak; ^3^ potential of the cathodic peak; ^4^ current of the cathodic peak; ^5^ ΔE = Epa − Epc; ^6^ E_1/2_ = (Epa + Epc)/2.

**Table 2:** Equation of linear dependence between I_pc_ and c, R^2^ (*n* = 19), LOD and LOQ for catechin at a CNT-Lac/SPE, GNP-Lac/SPE and CNT-GNP-Lac/SPE based on differential pulse voltammetric detection.

Electrode	Equation	R^2^	LOD (M)	LOQ (M)
CNT-Lac/SPE	Ipc = −1.61c–16.23	0.991	5.58 × 10^−8^	1.86 × 10^−7^
GNP-Lac/SPE	Ipc = −0.92c–9.70	0.990	1.29 × 10^−7^	4.30 × 10^−7^
CNT-GNP-Lac/SPE	Ipc = −1.06c–8.63	0.993	4.89 × 10^−8^	1.63 × 10^−7^

**Table 3:** Analytical performance of CNT-GNP-Lac/SPE compared to other sensors used for catechin detection, previously reported in the literature.

Sensors	Method	LOD (M)	Real-Life Samples	References
TAT-based polyimide-modified electrode ^1^	DPV	1.52 × 10^−5^	Green tea	[11]
(NiFe_2_O_4_/CoFe_2_O_4_/NCDs/MIP/GCE ^2^	DPV	1.3 × 10^−9^	Green tea	[77]
NG-Au@Pt NPs/Au electrode ^3^	DPV	2.8 × 10^−9^	Tea	[78]
SWCNT-SubPc/GCE ^4^	DPV	1.3 × 10^−8^	Tea	[79]
AgNWs-Tyr Modified Electrode ^5^	DPV	2.7 × 10^−6^	Red wine	[80]
CNT-GNP-Lac/SPE	DPV	4.89 × 10^−8^	Nutraceutical formulations containing green tea extracts	This study

^1^ 2, 4, 6-triamino−1,3,5-triazine-based polyimide-modified electrode; ^2^ dual-template molecularly imprinted polymer based on N-doped carbon dots incorporated into magnetic nanoparticle shell modified glassy carbon electrode; ^3^ N-doped graphene-Au@Pt core-shell nanoparticles/Au electrode; ^4^ single walled carbon nanotubes- subphthalocyanine glassy carbon electrode; ^5^ tyrosinase immobilized onto silver nanowires modified electrode.

**Table 4:** Characteristic parameters obtained with the three biosensors.

Biosensor	*I_max_*/µA	$K_{M}^{app}$/µM
CNT-Lac/SPE	−12.61	0.282 ± 0.007
GNP-Lac/SPE	−14.10	0.299 ± 0.009
CNT-GNP-Lac/SPE	−23.47	0.269 ± 0.004

**Table 5:** The results obtained by the CNT-GNP-Lac/SPE biosensor by interpolation in the calibration plot using DPV as voltammetric method, regarding the amount of catechin in the selected nutraceuticals, compared to those mentioned by the manufacturer on the label.

Nutraceutical Product	Catechin Content Specified by Manufacturer (mg/Capsule)	The Amount of Catechin Determined by DPV Method (mg/Capsule)
Green Tea Adams Vision	-	31.90 ± 1.32
Green Tea Extract Bio Synergie	15	16.80 ± 0.91
Green Tea Extract Zenyth	200	203.40 ± 4.07

**Table 6:** Antioxidant activity of catechin 10^−3^ M.

	DPPH	Galvinoxyl	ABTS
% Inhibition	60.77	49.70	75.85

**Table 7:** Antioxidant activity of the studied nutraceutical formulations.

Nutraceutical Product	% Inhibition-DPPH	% Inhibition-Galvinoxyl	% Inhibition-ABTS
Green Tea Adams Vision	19.36	19.25	31.07
Green Tea Extract Bio Synergie	18.34	18.99	31.22
Green Tea Extract Zenyth	70.63	70.65	95.70

**Table 8:** The values of the parameters obtained from the cyclic voltammograms of all three nutraceutical products recorded with CNT-GNP-Lac/SPE biosensor at a scan rate of 0.1 V·s^−1^.

Nutraceutical Product	Epa (V)	Ipa (µA)	Q400 (µC)
Green Tea Adams Vision	0.375	8.20	2.27
Green Tea Extract Bio Synergie	0.388	4.85	0.90
Green Tea Extract Zenyth	0.351	12.42	6.18

**Table 9:** Results of the electrochemical index of catechin and of the three nutraceuticals studied.

	Catechin	Green Tea Adams Vision	Green Tea Extract Bio Synergie	Green Tea Extract Zenyth
EI (µA/mV)	0.76	0.72	0.71	0.78

## Data Availability

The authors confirm that the data supporting the findings of this study are available within the article.

## Supplementary Materials

The following supporting information can be downloaded at: https://www.mdpi.com/article/10.3390/ijms23158110/s1.
