# Supplementary material for: Assessment of the Antioxidant Activity of Catechin in Nutraceuticals: Comparison between a Newly Developed Electrochemical Method and Spectrophotometric Methods
Source: Int J Mol Sci. 2022 Jul 23;23(15):8110. doi: 10.3390/ijms23158110 (PMC9329966; doi:10.3390/ijms23158110)
Supplement: Supplementary file 1 [file ijms-23-08110-s001.zip › ijms-1784512-supplementary.pdf]

Supplementary Material

Assessment of the Antioxidant Activity of Catechin in Nutraceuticals. Comparison between a Newly Developed Electrochemical Method and Spectrophotometric Methods

Irina Georgiana Munteanu and Constantin Apetrei*

Department of Chemistry, Physics and Environment, Faculty of Sciences and Environment, "Dunărea de Jos" University of Galaţi, 47 Domneasca Street, 800008 Galaţi, Romania; e-mail: georgiana.munteanu@ugal.ro, apetreic@ugal.ro.

* Correspondence: apetreic@ugal.ro; Tel.: +40-727-580-914

Supplementary information

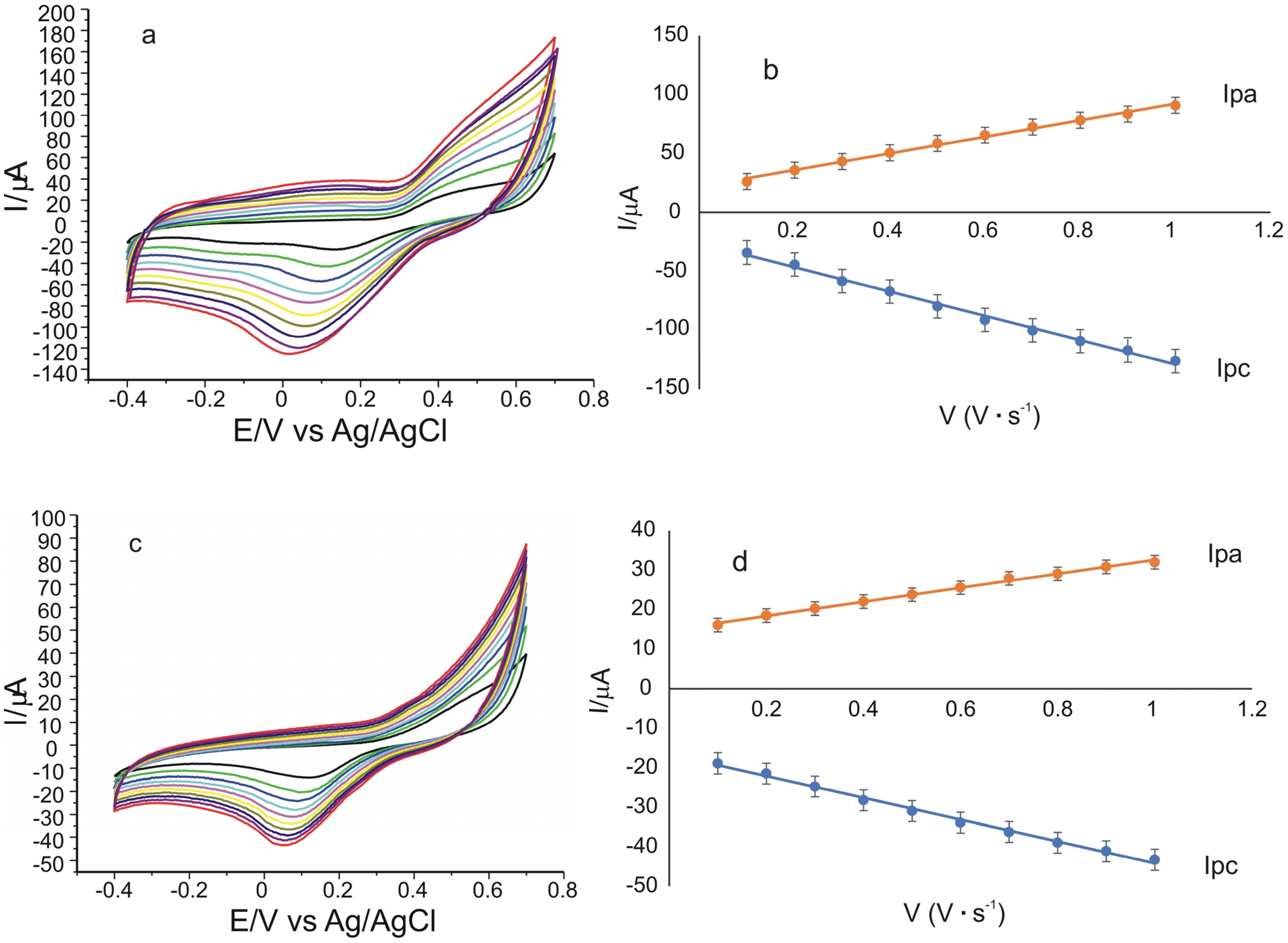

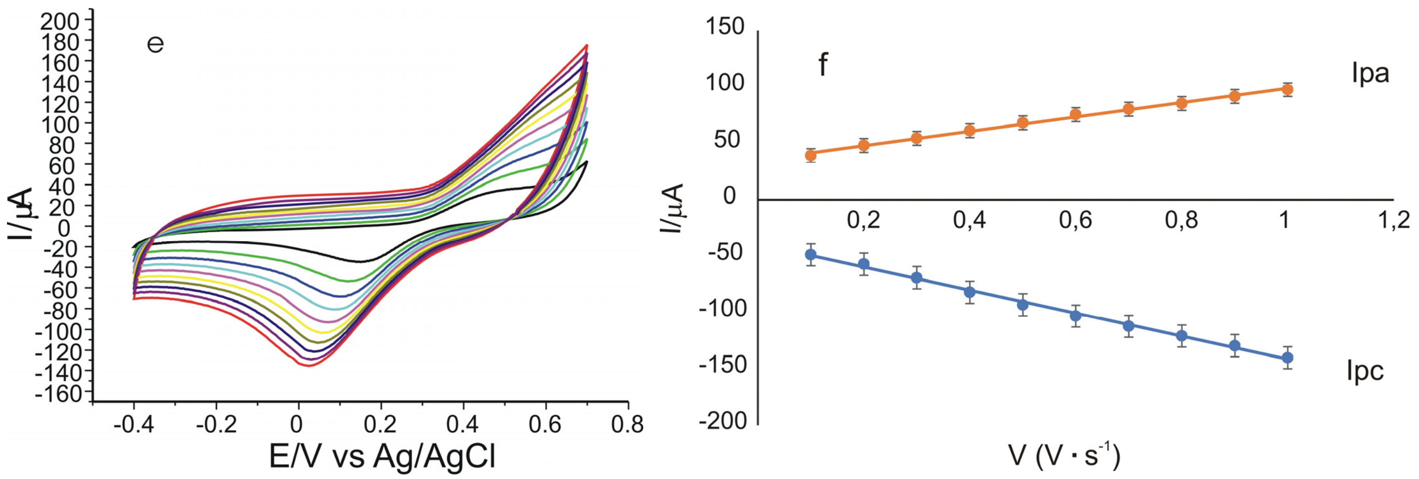

Figure S1. Cyclic voltammograms of CNT-Lac/SPE (a), GNP-Lac/SPE (c), CNT-GNP-Lac/SPE (e) immersed in 10^{-3} M catechin - 10^{-1} M acetate buffer (pH 5.2), recorded at various scan rates within the range 0.1–1.0 V·s^{-1}. Linear dependence between cathodic (Ipc) and anodic (Ipa) peak currents and the scan rates for CNT-Lac/SPE (b), GNP-Lac/SPE (d), CNT-GNP-Lac/SPE (f). Linear regression equations are: y = 70.43x + 21.63 (R^2 = 0.996, n=10) and y = -103.54x - 25.89 (R^2 = 0.995, n=10) for (b);

y = 17.79x + 14.8 (R^2 = 0.996, n=10) and y = -27.64x - 16.59 (R^2 = 0.995, n=10) for (d);

y = 63.02x + 35.73 (R^2 = 0.9955, n=10) and y = -102.33x - 39.16 (R^2 = 0.996, n=10) for (f).
